# Isotope Analysis Reveals Foraging Area Dichotomy for Atlantic Leatherback Turtles

**DOI:** 10.1371/journal.pone.0001845

**Published:** 2008-03-26

**Authors:** Stéphane Caut, Elodie Guirlet, Elena Angulo, Krishna Das, Marc Girondot

**Affiliations:** 1 Laboratory for Oceanology, MARE Center, B6C Liège University, Sart-Tilman, Liège, Belgique; 2 Laboratoire d'Ecologie, Systématique et Evolution, Université Paris Sud, Orsay, France; 3 Département Evolution et Systématique, Muséum National d'Histoire Naturelle de Paris, Paris, France; University of Pretoria, South Africa

## Abstract

**Background:**

The leatherback turtle (*Dermochelys coriacea*) has undergone a dramatic decline over the last 25 years, and this is believed to be primarily the result of mortality associated with fisheries bycatch followed by egg and nesting female harvest. Atlantic leatherback turtles undertake long migrations across ocean basins from subtropical and tropical nesting beaches to productive frontal areas. Migration between two nesting seasons can last 2 or 3 years, a time period termed the remigration interval (RI). Recent satellite transmitter data revealed that Atlantic leatherbacks follow two major dispersion patterns after nesting season, through the North Gulf Stream area or more eastward across the North Equatorial Current. However, information on the whole RI is lacking, precluding the accurate identification of feeding areas where conservation measures may need to be applied.

**Methodology/Principal Findings:**

Using stable isotopes as dietary tracers we determined the characteristics of feeding grounds of leatherback females nesting in French Guiana. During migration, 3-year RI females differed from 2-year RI females in their isotope values, implying differences in their choice of feeding habitats (offshore *vs.* more coastal) and foraging latitude (North Atlantic *vs.* West African coasts, respectively). Egg-yolk and blood isotope values are correlated in nesting females, indicating that egg analysis is a useful tool for assessing isotope values in these turtles, including adults when not available.

**Conclusions/Significance:**

Our results complement previous data on turtle movements during the first year following the nesting season, integrating the diet consumed during the year before nesting. We suggest that the French Guiana leatherback population segregates into two distinct isotopic groupings, and highlight the urgent need to determine the feeding habitats of the turtle in the Atlantic in order to protect this species from incidental take by commercial fisheries. Our results also emphasize the use of eggs, a less-invasive sampling material than blood, to assess isotopic data and feeding habits for adult female leatherbacks.

## Introduction

Yalimapo beach in French Guiana, South America, is one of the main nesting grounds for the largest of the sea turtles, the leatherback (*Dermochelys coriacea*), visited by up to 30–40% of the world's population of nesting females [1]. The major nesting season at Yalimapo extends from March to the end of August, including a peak in June, with some sporadic nesting outside this period [2]. During the nesting season a female may lay as many as 14 clutches, with an average interval between nesting events of 10 days [1]. Due to the segregation of breeding and foraging sites, at the end of the reproductive season leatherbacks migrate several thousand kilometers [3], [4] across ocean basins to pelagic foraging areas where their prey, large jellyfish and other gelatinous organisms, is more abundant than on tropical coasts [5]–[7]. The leatherback is the only marine turtle that feeds on gelatinous zooplankton throughout its life. This diet is extremely poor in lipids and energy, and consequently very little energy and nutrient is extracted from a given mass of prey [5]. Davenport [5] estimated that leatherbacks in cold sea water (e.g. North Atlantic) consume a quantity of prey equivalent to at least 50% of their body mass per day.

During the breeding season, energy requirements are high because of egg production, nest construction, and swimming activity between nesting events [8]. However, leatherbacks appear to adopt different strategies during their inter-nesting intervals, depending on their nesting site [9]. In the Eastern Pacific Ocean (e.g. Costa Rica and Mexico rookeries) leatherbacks may minimize energy expenditure to maximize the amount of energy allocated to ovipositing and egg production [8], [10]. In the Atlantic Ocean and Western Pacific Ocean (Indonesia rookery) leatherbacks cover great distances at high speed and disperse extensively, probably for feeding [9], [11]–[14]. However, the majority of studies on the feeding ecology of leatherback turtles have been based on dive patterns during inter-nesting intervals, and not on diet analysis. Diving activity during the migration cycle is closely related to foraging activity on pelagic prey [15], whereas diving during inter-nesting intervals is less well understood [16]. Hays *et al.* [6] showed that dive duration varied with foraging success; dives were much longer in feeding areas, where foraging success was higher, than along tropical coasts, which provided limited foraging opportunities.

The time between successive nesting seasons for a female is called the remigration interval (RI). The RI for leatherback turtles is variable, but is most commonly 2 or 3 years [17]. The reasons for variation in the RI are unknown [18]. It seems that female turtles require a specific level of energy reserves prior to migration to nesting beaches, in order to undergo vitellogenesis and nest successfully [19]–[21]. They may delay reproductive migration until a reproductive energy reserve threshold is reached [22]. Availability of nutrients, particularly in aquatic systems, has been shown to be affected by climatic oscillations such as the El Nino Southern Oscillation (ENSO) and the North Atlantic Oscillation (NAO) [18], [21]. This implies that abundance and distribution of gelatinous prey for leatherbacks are spatially and temporally unpredictable [18]. Thus, the RI would depend on the capacity of breeding turtles to find favorable foraging areas.

Recent satellite transmitter data has revealed that Atlantic leatherbacks follow two major dispersion patterns after nesting, either through the North Atlantic area or more easterly at low latitudes across the North Equatorial Current [3], [4], [23]. It has been suggested that the RI could be heavily influenced by ecological conditions in turtle foraging areas, such as surface temperature or trophic status [24], [25]. It may be that dispersion patterns are linked to the RI of female turtles, with varying ecological conditions and varying proximity of foraging areas to nesting beaches determining the specific RI for each foraging area.

Individuals that exploit geochemically different habitats, or feed on different resources, can be differentiated using stable isotope measurements, as the isotope profile of consumers reflects that of their prey. This approach is based on the fact that stable isotope ratios of nitrogen (^15^N/^14^N, noted δ^15^N) and carbon (^13^C/^12^C, noted δ^13^C) in the consumer tissues reflect those in their resources in a predictable manner due to selectivity for lighter isotopes during a consumer's metabolic processes [26], [27]. The difference between isotopic values of consumer and their preys, called discrimination factor, vary among tissues and taxa, but is often between 0–1‰ for δ^13^C, and 3–4‰ for δ^15^N [26]–[28]. Also, isotope analysis offers advantages over traditional methods (e.g. direct observation of feeding behavior, gut content analysis) because it provides time-integrated information on foods assimilated. The period of time over which the tissue isotope values of a consumer reflect the values of their diet is called the turnover rate. Tissues such as liver and plasma have high turnover rates that reflect recent diet, whereas tissues with slower turnover rates, such as blood cells and muscle, reflect diet over longer periods [29], [30]. Stable isotope measurements have also been used to infer estimates of trophic level and animal movement patterns for both invertebrates and higher vertebrates [31]. Consumers are typically enriched in ^15^N relative to their food and consequently δ^15^N measurements serve as indicators of a consumer's trophic position [32], [33]. By contrast, δ^13^C values vary little along the food chain and are mainly used to determine primary sources in a trophic network [32], [34]. In the marine environment, the δ^13^C values can also indicate inshore versus offshore, or pelagic versus benthic contribution to food intake [35]–[39]. This difference may be related to the tendency of δ^13^C values to decrease from low to high latitudes, due to oceanographic factors such as CO_2_ concentration effects on carbon fixation by phytoplankton [40], [41].

The use of stable isotope has become a powerful tool for clarifying questions about nutritional ecology and migration of marine vertebrates [42]. However, relative to other taxa, there is a paucity of stable isotope studies in marine turtles. The first was Godley *et al.* [43] that determined trophic status of marines turtles from the Mediterranean Sea and the European Atlantic Ocean. Since then, analyses of isotopes were used to study respiratory physiology [44], feeding habitat in Japan [45], [46], western Mediterranean [47], [48] and bahamas [49] and trophic dichotomy between ocean basins [41].

In this paper we present the first isotopic analysis of leatherback turtle nesting in French Guiana and inferred from isotopic leatherback data collected several times on the same females during a nesting season. We hypothesized that females with different RIs forage at different locations, and differ in the isotopic values of the tissues subject to a low turnover (Red Blood Cells-RBC). Our hypothesis predicted that females with 3 and 2 year RI exploit geochemically different habitats varying in latitude (North Atlantic *vs.* West African coast) and/or origin of carbon source (offshore *vs*. coastal). We also tried to investigate whether females forage in the breeding areas. Finally, we examined the relationship between stable isotope values of eggs and female tissues to assess egg analysis as a less-invasive method of sample collection for isotope measurements in this endangered marine turtle.

## Results

From March to May 2006, we sampled 52 leatherback females at Yalimapo beach in French Guiana: 1 female sampled for 5 different clutches (interval between first and last clutch: 44 d), 7 females for 4 clutches (minimum and maximum interval between the first and the last clutch: 27–49 d), 1 female for 3 clutches (40 d), 14 females for 2 clutches (9–43 d) and 29 females for 1 clutch. The different clutches were not necessarily consecutive; For example, a female that was sampled for 4 clutches during 49 days laid 6 clutches during this period, but 2 were not sampled. Among the turtles 23 had been tagged and recorded in a passive integrated transponder tags (PIT) database, which showed that 7 had a 3-year RI and 16 had a 2-year RI.

The curved carapace length (CCL) of sampled females ranged from 142–172 cm (mean±SE = 160±1 cm, n = 48). A preliminary analysis showed that CCL had no effect on δ^13^C and δ^15^N in any tissue (*P*>0.5).

Two eggs were sampled from each of three clutches to assess intra-clutch variability in isotope values. Differences between the values for the 2 eggs within a clutch were of the same order as the measurement error, ranging from 0.10–0.15‰ for δ^13^C and 0.07–0.15‰ for δ^15^N. Despite the small sample size, we assumed that a single egg-yolk reflected the δ^13^C and δ^15^N of the whole clutch.

### Do females forage during the breeding season?

**Figure 1:**
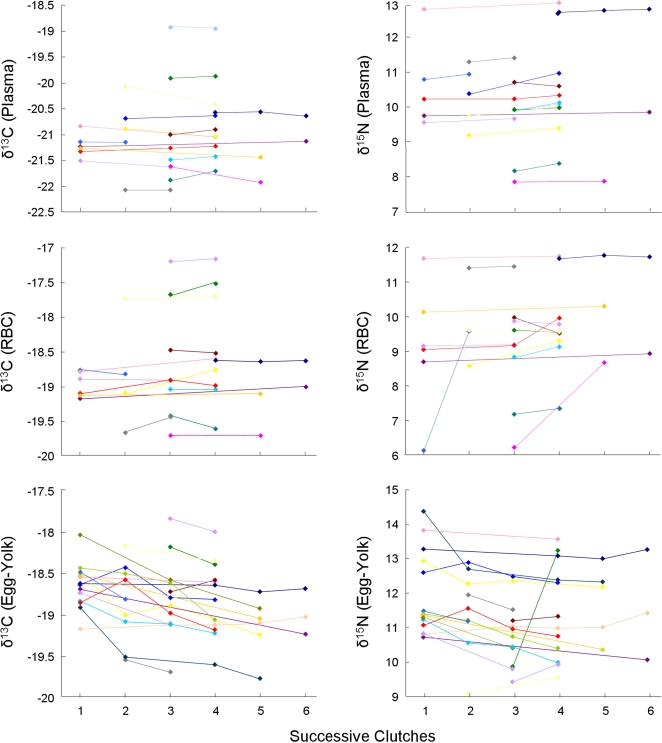
Trends in isotopic values for δ^13^C and δ^15^N in plasma, RBC, and egg-yolk of nesting leatherback turtles. Each colour represents data for the successive clutches laid by one female; each sampled clutch is represented by a point.

We tested whether tissue isotope ratios varied over the nesting period by performing general linear models, with repeated measures of δ^13^C and δ^15^N for each tissue in relation to the time (in days) of each clutch after the first clutch was observed. The number of eggs in each clutch was included as a covariable, but had no effect on δ^13^C and δ^15^N for any tissues (*P*>0.2 in all cases). The time of each laying event had no effect on δ^15^N values for any tissue (Table 1A). Time also had no effect on the δ^13^C value for RBC and plasma, but it had a significant effect on the δ^13^C value for egg-yolk (Table 1A). δ^13^C values in egg-yolk decreased significantly from one clutch to the next. Thus, except for egg-yolk δ^13^C values, isotope values of females did not change significantly over the breeding season (Fig. 1).

**Table 1:** Variations of isotopic values of three different tissues.

	Dependent variables→		δ^13^C		δ^15^N	
	Independent Variables↓	*N*	F/χ2	P	F/χ2	P
**A**	Egg-yolk	*81*				
	Number of eggs		0.60	0.437	0.15	0.702
	Days between nesting		6.15	***0.013***	0.17	0.681
	Plasma	*63*				
	Number of eggs		0.11	0.736	0.87	0.352
	Days between nesting		0.06	0.800	1.51	0.219
	RBC	*63*				
	Number of eggs		1.29	0.256	0.20	0.653
	Days between nesting		0.00	0.990	2.95	0.086
**B**	Egg-yolk	40				
	Remigration interval		2.10	0.156	0.57	0.453
	Plasma	*31*				
	Remigration interval		4.25	0.054	<0.01	0.989
	RBC	*31*				
	Remigration interval		10.22	***<0.01***	<0.01	0.973
**C**	Egg-yolk	*50*				
	Plasma		122.27	***<0.001***	178.60	***<0.001***
	Regression equation		δ^13^C_P_ = 4.66+1.37δ^13^C_Egg_ (R^2^ = 0.84)		δ^15^N_P_ = 1.58+0.81δ^15^N_Egg_ (R^2^ = 0.89)	
	RBC		138,3	***<0.001***	24.54	***<0.001***
	Regression equation		δ^13^C_RBC_ = 3.72+1.19δ^13^C_Egg_ (R^2^ = 0.86)		δ^15^N_RBC_ = 2.76+0.64δ^15^N_Egg_ (R^2^ = 0.64)	

Variations with (A) the time in days between nesting events and the number of yolked-eggs and (B) the remigration interval. (C) Relationships between egg-yolk isotopic ratios and RBC and plasma isotopic ratios. Significant results are marked in bold.

The mean (±SD) isotope values of the whole body of the jellyfish sampled at the beach during the breeding season were very high for carbon and similar for nitrogen (−15.8‰±1 and 9.3‰±1.4, *n* = 5, respectively) compared with the values for blood (RBC: −18.8‰±0.1 and 9.5‰±0.2, plasma: −21.1‰±0.1 and 10.2‰±0.2, *n* = 63, respectively) and for egg-yolk (−18.83‰±0.42 and 11.16‰±1.26, *n* = 81).

### Do females with 2-year and 3-year RIs differ in their isotope values?

**Figure 2:**
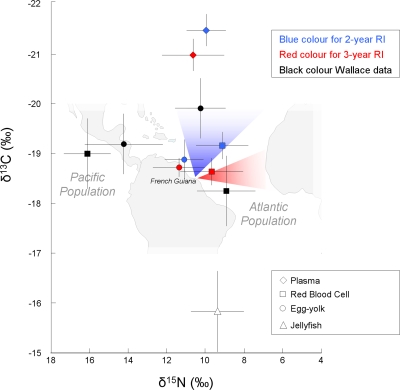
Effect of remigration interval (RI) on δ^13^C and δ^15^N leatherback tissus (plasma, RBC and egg-yolk). Values are mean±SD. The black symbols represent the values of Wallace *et al.* [41] for Atlantic and Pacific populations of leatherback turtles, provided for comparison. The map shows the two major patterns of migration of Atlantic turtles, from nesting beaches to foraging areas, following Ferraroli *et al.* [3] and Hays *et al.* [23].

Females with 2-year and 3-year RIs differed significantly in the δ^13^C values in RBC. In plasma the difference between the two groups was nearly significant for δ^13^C (Table 1B). However, δ^15^N values in RBC and plasma remained similar between turtles with 2-year and 3-year RIs (Table 1B, Fig. 2). Isotopic values (δ^13^C and δ^15^N) of egg-yolk were not different between females with 2-year and 3-year RIs (Table 1B).

### Relationship between egg-yolk and female blood isotope values

Egg-yolk δ^13^C and δ^15^N were positively correlated with the corresponding RBC and plasma δ^13^C and δ^15^N values for females (Table 1C).

## Discussion

### Do females forage during the breeding season?

Several studies using techniques such as video recording, mouth sensors and depth recorders have failed to adequately elucidate the feeding habits of leatherback turtles during the nesting season [10], [12], [16]. Diving patterns tended to show that Atlantic leatherback females feed during internesting intervals [9], [13], and foraging was suggested in southern Caribbean females (although with low success, [12]). Conversely, video recording showed no foraging during the breeding season in Costa Rica [10]. However, with one exception in a foraging area (in the north-west Atlantic [50]), no study has involved diet analysis because this species is difficult to study.

The use of stable isotope ratios has become a powerful tool to estimate and clarify the feeding ecology of many vertebrate, especially when species are particularly difficult to study by traditional methods [31]. The stable isotope ratios can provide new insights into the foraging activity of females during the breeding season, but measuring turnover rate and discrimination factor (the isotopic difference between the animal and its food source) in turtles'tissue is a necessary process to enable a correct interpretation. Stable isotope values for plasma have been shown to represent more recent dietary resources in bears [51]. However, there is a lack of information on plasma turnover rates for turtles. Seminoff *et al.* [52] compared plasma values in green sea turtles (*Chelonia mydas*) at 371 and 614 days in a captivity study, and found that turnover was at least 371 days. However they did not show the dynamic of the turnover. Seminoff *et al.* [53] estimated that nitrogen turnover rate in plasma of freshwater turtles, *Trachemys scripta*, was 142 d increasing linearly, with a half-life (the time in which half of the stable isotopes were exchanged in the tissue) of 35.6 days. Based on these unique data on turtles and knowing that the longest nesting season observed during our study for a leatherback female was 49 days, we can suppose that isotopic turnover of plasma in the leatherback turtles sampled may have not been completed, but that a change in isotopic values could have been detected. However, no significant variation in plasma δ^13^C and δ^15^N throughout the breeding season was detected.

Parallel measures of isotope values in jellyfish sampled on the nesting beach did not indicate food intake near the beach. Massive strandings of two jellyfish genera (*Rhizostoma* and *Aurelia*) are regularly observed on Yalimapo beach, and they are known to be a common prey of leatherback turtles [50]. If turtles had eaten and begun to assimilate this prey, the isotopic value of plasma would have been −15.9‰ and 12.2‰ for carbon and nitrogen respectively (based on discrimination factors estimated on green turtle [52]). Even if the turnover was not reached, the time frame of our study (a maximum interval of 49 d) should have allowed a change in isotopic values as it represents the half-time of turnover in plasma of emydid turtle [53]. During this interval, emydid turtles increased 0.9‰ and 2.4‰ for carbon and nitrogen respectively, while leatherbacks increased only 0.1‰ and 0.11‰; this result seems to indicate that leatherbacks did not feed during this period.

A further complicating factor to interpret isotopic data is the nutritional stress [34], [54]. It is generally accepted that tissues of fasting animals become enriched in δ^15^N following nutritional restriction [54]–[56]. Hobson and Clark [57] explained that increases in diet-tissue fractionation values are due to mobilization and redeposition of proteins elsewhere in the body or amino acid composition changes in tissues. Following this argument, it could be expected δ^15^N in mobilized organic molecules to be higher in comparison with the initial state of the organic molecules in the body reserves. Voigt and Matt [58] gave another possible explanation: the different extent of metabolic processing of two nitrogen sources, i.e. internal body reserves (fat) and external food sources (carbohydrates), may lead to different nitrogen enrichments. However, our inability to detect such an increase may also be due to the species of study. Hobson and Clark [57], as well as Hobson *et al.* [54], documented an increase in δ^15^N values with nutritional stress in several avian species. Kempster *et al.* [59] found that a 35% reduction in food intake had no effect on δ^15^N values of tissues in song sparrows (*Melospiza melodia*). Williams *et al.* [60] found whole blood and blood cells from nutritionally restricted tufted puffin (*Fratercula cirrhata*) nestlings were significantly depleted in ^15^N compared to well-fed conspecifics. Thus, the effect of nutritional restriction could be dependent of species-specific differences in physiological response [60] or largely a function of the level of nutritional stress [59].

While past experiments on mammals, birds, fishes, and insects have shown changes in stable isotope ratios due to nutritional stress, there has been no research on this topic in reptiles. The detection of nutritional stress in wild population is very difficult compared to experimental studies. Indeed, the short timeframe of our samples in relation to the slow turnover rate of turtle blood, the particular physiological characteristic of the endothermic capacity of leatherbacks [5], the relatively low metabolic rates between nesting events [8] and the few data on foraging at this period [9], [10] make it difficult to establish a conclusion concerning fasting or foraging activity of females during nesting season [53]. Based on these considerations, it is obvious that this point would benefit from further studies on the isotopic values of sea turtles.

### Remigration intervals and foraging sites

Leatherback turtles undertake long migrations between nesting sites and foraging grounds. The causes of variation in the RI of female leatherbacks are unknown, but may be linked to the availability of food sources and thus the ability to store enough energy resources to undertake migration to breeding areas [25]. Stable isotope analyses can offer additional information on feeding strategies or movement patterns of migratory species [31], [34], [61]. The stable isotope composition of an organism depends on the diet source and also on the isotopic signature at the base of the food web [26], [27], [62]. Indeed, different oceanic processes affect isotopic baselines of δ^15^N and δ^13^C [61]; spatial and temporal variability has been shown to be incorporated and conserved through several trophic levels across ocean basins or within a region of a single basin [41], [56], [63]. Hobson [42] illustrated this approach by the new maxim “you are what you swim in” that complements the well-known dogma of stable isotope ecology “you are what you eat” [64]. Consequently, the stable isotope ratios of animal tissues have the potential of characterizing isotopically distinct regions. 

Leatherback females in French Guiana (2-year and 3-year RI) did not differ in their δ^15^N values. Wallace *et al.* [41] showed that δ^15^N in leatherback turtles indicates oceanographic differences, as the distinct nitrogen cycling regimes among oceans influences the baseline δ^15^N signatures of marine food webs [41]. Thus, the δ^15^N values of egg yolk and RBC were significantly different between an Atlantic population (St Croix) and a Pacific population (Costa Rica, more enriched by 4.5 and 7.2‰, respectively). In this sense the δ^15^N values for French Guiana turtles were more similar to the St Croix population (Fig. 2). The turtles in our study had a broad range of δ^15^N values (e.g. δ^15^N for RBC = 6.1–12.3‰), which probably reflects different feeding strategies on gelatinous zooplankton: leatherbacks are known to feed on planktivorous jellyfish (*Rhizostoma octopus*, *R. pulmo*, *Aurelia aurita, Stomolophus meleagris*) or jellyfish that forage on fish or crustaceans (*Cyanea capillata*, *C. lamarckii*, *Pelagia noctiluca*) [65].

Although individual females may show a degree of subregional fidelity to areas such as northward to the Gulf Stream area or eastward to tropical waters [3], [23], Atlantic leatherbacks could disperse widely across most of the ocean basin but have not yet been tracked across the equator [66]. During their remigration interval, several foraging grounds have been identified: North Atlantic ocean as Scotia Nova, East Costs of USA, Ireland costs, Bay of Biscay or more southern in West African and Iberian coasts [9], [11], [19], [50], [67]–[69]. However, two main migration patterns have been showed: northward to the Gulf Stream area or eastward to tropical waters [3]. We hypothesized that these patterns could be linked to different ecological foraging areas, and that this could explain differences in RI between females foraging in those areas. C isotope ratios are conservative from phytoplankton up to top consumers with less than 1‰ enrichment per trophic level [62] and hence are ideal to trace gradients in the marine environment, as the signature of a consumer should reflect the sources of C at the base of the food chain. The δ^13^C values in the tissue of marine animals have been shown to vary with latitude (e.g. [34], [63]), reflecting the depletion of ^13^C in phytoplankton towards higher latitudes [61], [70]. Moreover, the general pattern of inshore, benthically linked food webs being more enriched in ^13^C compared with onshore, pelagic food webs presents a potentially useful tool for marine biologists. Also, stable isotope analysis has increasingly been used to infer animal movement patterns from invertebrate to higher vertebrate (see reviews, [31], [34], [42]). Differences in δ^13^C blood values between 2-year and 3-year RI females suggest that the French Guiana leatherback population segregates into two distinct isotopic groupings, and seem to confirm that RI is linked to foraging areas. As isotope signatures could provide information on both the latitude, and the pelagic *vs.* neritic nature of the foraging ground, it is possible to reveal foraging area dichotomy for 2-year and 3-year RIs. The low RBC δ^13^C values in the 2-year RI turtles suggests that the C resource of these turtles is situated in a more northern and/or offshore region (high latitude in North Atlantic), and the high values of the 3-year RI turtles indicate a more southern and/or coastal foraging area (West African and Iberian coasts). Confirming these conclusions, maps of gelatinous organism distribution provide information on prey location and indicate that these foraging areas (central North Atlantic and west coasts of Africa) support several appreciable aggregations of potential prey, especially due to the presence of upwelling and frontal areas [11], [71]. Moreover, leatherbacks are generally considered completely pelagic, but are also observed in coastal waters when food is available [11], [72].

### Use of egg yolk isotope signatures

Stable isotope ratios in the diet become incorporated into egg yolk in 8–15 days in birds [73], but could require more time in reptiles due to metabolic differences. Female leatherbacks arriving at the nesting beach carry a full complement of follicles to supply yolks for all the eggs that will be laid during the season [74]. Vitellogenesis probably lasts 3–6 months and is complete upon arrival at nesting sites before mating begins [75]. Thus, the energy and chemical components contained in the follicles were derived from food eaten in the foraging area [74]. Hobson [73] showed in birds that following a change of diet the isotope signature of yolk was proportional to the additional mass of yolk formed from the new diet. The situation in sea turtles is more complex. Before each nesting event yolk is formed from dietary sources during rapid follicle growth, and so could reflect the contribution of a new diet to isotope values. In our study, the δ^15^N values in egg yolks were not significantly different among serial clutches from one turtle, but the δ^13^C values were significantly different and tended to decrease from one clutch to another. No explanation for these different trends is apparent. If females had fed on jellyfish at Yalimapo beach and incorporated this C source into egg yolk, the trend of egg yolk δ^13^C values should have been reversed. Hatase *et al.* [46] found opposite results in green turtles (*Chelonia mydas*), with δ^13^C not significantly different and a significant enrichment in δ^15^N egg yolk values among five serial clutches from one turtle. This enrichment could not be attributed to nutritional stress from fasting [46]. Hatase *et al.* [45] found no significant difference in δ^13^C and δ^15^N in egg yolks among four serial clutches of a single loggerhead turtle.

We found a positive relationship between isotope values (δ^13^C and δ^15^N) of the blood of females and their egg yolks. This indicates that stable isotope analysis of egg components is a viable method for assessing foraging ecological questions in marine turtles. Thus, to study the diet of adult sea turtles, stomach lavage or blood sampling during laying could be replaced by a procedure less-invasive, sampling the yolk of a single egg.

### Management and conservation significance

Establishing patterns of movements of free-ranging animals is crucial for a better understanding of their feeding ecology and life history traits, and is a prerequisite for their conservation. Tracking animal movements can be done directly using remote-sensing techniques or indirectly using biochemical markers like naturally occurring stable isotopes. This method is less-invasive, repeatable and can be applied over different time scales to investigate migration or feeding ecology, and is very appropriate to the study of endangered species such as sea turtles. Stable isotope analysis in sea turtle can also be used to assess the feeding ecology and habits in inaccessible locations. For example, Reich *et al.* [49] identified habits and diet of the cryptic juvenile life stage of the green turtle. However, studies using stable isotope to infer foraging location are few. In the present study, stable isotopic values combined with telemetry data of literature were used to improve knowledge on the foraging areas used by the two remigration groups of females. Unfortunately, the theoretical and experimental basis of this method remains poorly validated for sea turtles, but is essential for the correct interpretation of field data. The first study, by Seminoff *et al.* [52], of *Chelonia mydas* maintained in a controlled environment is very encouraging. However, although the use of stable isotope analysis has a number of advantages in food web and migration studies, the optimal approach is to combine it with direct observation of feeding behaviour (or stomach analysis on dead female), which provides a taxonomic resolution of resources, and satellite telemetry, which provides migration travels [42], [45].

We underline two important points for the conservation of leatherback turtles. Firstly, two foraging area dichotomy for Atlantic leatherback turtles are used by 2-year and 3-year RI females. Foraging in the more southern and coastal area of West Africa seems to delay the return of females to breeding areas by one year. More research is needed to understand whether females always select the same foraging areas, and if so what the evolutionary benefits are in choosing a particular foraging site (e.g. it would be very interesting to compare isotopic values in tissues from the same individual between seasons). Fisheries bycatch is believed to be among the primary causes of leatherback turtle decline followed by egg and female harvest [76], [77]. Recent research has shown that small-scale fisheries, which operate near coasts, may be a greater threat to leatherback turtles than industrial-scale fisheries [78], and the conservation measures proposed by these authors are clearly needed in coastal foraging areas. This study responds to an aforementioned need to delineate the feeding habitats of the leatherback turtle in the Atlantic Ocean, to protect this species from incidental take in commercial fisheries. In addition, isotope values of eggs have been shown to reflect the values of female tissues, providing an effective and non-invasive method for study of this endangered reptile.

## Materials and Methods

### Study site and collection of samples

**Figure 3:**
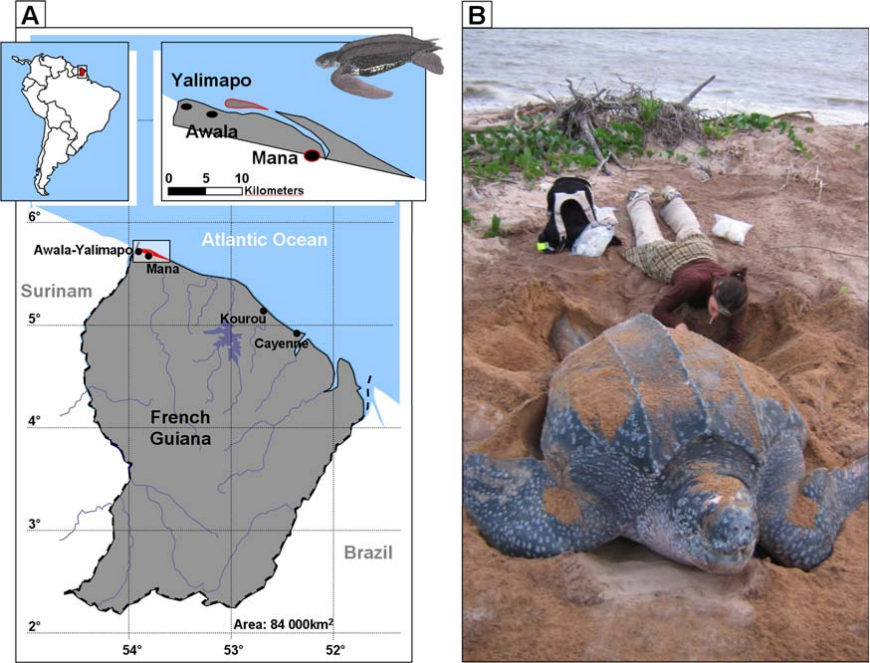
Location of the study site, and leatherback turtle blood sampling. A Map of Awala Yalimapo nesting beach in French Guiana. B Blood sampling of a large female leatherback turtle (*Dermochelis coriacea*) during a nesting event.

Research was carried out within the Amana Nature Reserve at Yalimapo beach in French Guiana (53°57′W, 5°45′N), on the inshore plain of the coastline between the Mana and Maroni Rivers (Fig. 3A). Leatherback turtles nesting on this beach are tagged with internal permanent markers (passive integrated transponder [PIT] tags) placed in the turtle's right shoulder muscle, which enables temporal monitoring of the females during and between nesting seasons. Since 1985 conservation workers on this beach have surveyed turtles almost every night during the main nesting season (mid-April to mid-July).

Eggs and blood were sampled nightly from turtles on Yalimapo nesting beaches, from 4 h before until 4 h after the high tide from the 16th March to 14th May, 2006. Nesting females were scanned for PIT tags. At the beginning of the nesting season all females were sampled. Sampling then focused on females that had already been captured one or more times. The curved carapace length (CCL) was measured to the nearest cm as an index of size. The number of eggs in each clutch was recorded and one fertile egg was collected. For three females we collected two eggs laid successively, in order to examine intra-clutch variation in stable isotope ratios of the egg yolk. Blood was sampled in the venous sine of the rear flipper [79], using single-use syringes and blood collection tubes containing heparin to prevent clotting (Fig. 3B). In April and May we also collected jellyfish as potential prey of leatherbacks in the area; they strand regularly on Yalimapo beach. All samples were frozen at −20°C until analyzed.

### Stable isotope analysis

Samples of egg yolk and turtle blood (plasma and red blood cells–RBC, centrifugalized at 5200 rpm for 5 min), and whole specimens of jellyfish were used for isotope analyses. Lipids were previously removed from egg yolk with a dichloromethane-methanol (2∶1) solution. All samples were dried at 60°C for 48 h, ground to a fine powder, weighed in tin capsules and stored in a dessicator until isotope measurement. Isotope analyses were performed using an IsoPrime spectrometer (*MicroMass*, Service Central d'Analyse, Solaize, France) coupled to a EuroEA 3024 analyzer. Stable C and N isotope ratio are expressed as:
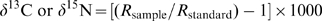
Where *R* is ^13^C/^12^C or ^15^N/^14^N for δ^13^C and δ^15^N, respectively. The standard for the C isotopic ratio is IAEA-NBS 21 (graphite −28.13‰), and for the N isotopic ratio is IAEA-N1 (+0.4‰) and IAEA-N2 (+20.3‰). Ten replicate assays of internal laboratory standards indicated measurement maximum errors (SD) of ±0.15‰ and ±0.2‰ for stable carbon and nitrogen isotope measurements, respectively.

### Hypothesis tested and statistical analysis

We firstly examined variation in isotope ratios in the plasma, RBCs and egg yolk of each female throughout the nesting season (between different laying events) in order to establish whether females forage during the breeding season. We carried out general linear models with repeated measures (repeated measures GLM) in which the dependent variable was each isotope ratio (δ^13^C and δ^15^N), and the main independent variable was the time in days of each clutch since the first clutch was observed (time 0 corresponded to the day when we observed the first clutch for each female). We added a covariable that was the number of eggs in each clutch. Repeated measurements enabled comparison of data from the same female at different laying events. The normality of the dependent variables was confirmed prior to the analyses.

We secondly searched for differences in RBC and plasma isotope values between 2-year and 3-year RI females. We carried out a general linear mixed model (GLMM) for each tissue and each isotopic ratio (δ^13^C and δ^15^N; the dependent variables of each model). We used mixed models because values for the same female at different times (representing different laying events) were correlated; this covariance structure was handled by introducing the individual females as a random effect into the GLMM.

Finally, to assess if female isotope ratios could be estimated from egg samples only, we tested the relationship between isotope ratios of egg yolk, and RBC and plasma from the same female. We performed independent GLMM in which the dependent variable was δ^13^C or δ^15^N for egg yolk and the independent variables were δ^13^C and δ^15^N of RBC or plasma. Individual females were introduced in the model as a random effect, as explained above. Because one of the goals of this analysis was to establish a method to obtain isotope ratios of females when female tissue samples are not available, we determined regression equations between egg yolk isotope ratios and those for RBC and plasma, through simple regression models when GLMM were significant.

All analyses were performed with the STATISTICA package, version 6.0 [80], and the SAS package, version 9.1.3 [81].
