# Correction: Isotope Analysis Reveals Foraging Area Dichotomy for Atlantic Leatherback Turtles

**DOI:** 10.1371/annotation/de853c89-d3eb-441f-8d87-0366bb1533b5

**Published:** 2009-07-16

**Authors:** Stéphane Caut, Sabrina Fossette, Elodie Guirlet, Elena Angulo, Krishna Das, Marc Girondot, Jean-Yves Georges

Drs. Sabrina Fossette and Jean-Yves Georges were not included in the author byline. They should be listed as the second and seventh authors, respectively, and affiliated with Institut Pluridisciplinaire Hubert Curien, Département Ecologie, Physiologie, Ethologie, UMR 7178 CNRS/Université de Strasbourg, Strasbourg, France. The citation should be: Caut S, Fossette S, Guirlet E, Angulo E, Das K, Girondot M, Georges J-Y (2008) Isotope Analysis Reveals Foraging Area Dichotomy for Atlantic Leatherback Turtles. PLoS ONE 3(3): e1845. doi:10.1371/journal.pone.0001845.

