# Correction: Isotope Analysis Reveals Foraging Area Dichotomy for Atlantic Leatherback Turtles

**DOI:** 10.1371/annotation/72a9b040-40d8-495a-a4aa-a7ea8fd00de8

**Published:** 2009-08-07

**Authors:** Stéphane Caut, Sabrina Fossette, Elodie Guirlet, Elena Angulo, Krishna Das, Marc Girondot, Jean-Yves Georges

Because two authors were added to the author byline, the funding statement should also include the following: SF was supported by a studentship from the French Ministry of Research. JYG was supported by Programme Amazonie du CNRS and ANR through Projet MIRETTE. 

