# Correction: Isotope Analysis Reveals Foraging Area Dichotomy for Atlantic Leatherback Turtles

**DOI:** 10.1371/annotation/17755b0c-3597-4da2-be87-08a14caba677

**Published:** 2009-08-12

**Authors:** Stéphane Caut, Sabrina Fossette, Elodie Guirlet, Elena Angulo, Krishna Das, Marc Girondot, Jean-Yves Georges

Because two authors were added to the author byline, the Author Contributions do not correctly reflect the new authorship. The "Conceived and designed the experiments" statement should now include the following: SF EG MG JYG. The "Performed the experiments" statement should now include the following: SC SF EG JYG.

